# Factors for innovation adoption by ports: a systematic literature review

**DOI:** 10.1007/s40722-024-00339-9

**Published:** 2024-08-21

**Authors:** Krishna Sooprayen, Geerten Van de Kaa, Jeroen F. J. Pruyn

**Affiliations:** Technische Universiteit Delft, Delft, The Netherlands

**Keywords:** Maritime, Ports, Innovation adoption, PRISMA, Dominant designs

## Abstract

This paper investigates the factors influencing innovation adoption in ports by conducting a systematic literature review and proposes a comprehensive framework for understanding the process of innovation adoption. The maritime sector is a typical example of a business-to-business market, whereas the information technology industry is an example of a business-to-consumer market. We show that factors for innovation adoption applicable to a business-to-consumer market are also relevant to a business-to-business market. The factors that were found relate to the adopting port’s characteristics and include know-how, organization support, organizational structure, financial capacity, a port’s network embeddedness, and risk-taking. Furthermore, they concern the characteristics of the innovation such as the costs, relative advantage, complexity, compatibility, trialability, and observability. Finally, stakeholder pressures were identified relating to the customer, competitive port, regulatory bodies, and society.

## Introduction

Almost 80% of the international trade of goods, in terms of ton-miles, is transported by sea vessels (UNCTAD 2021). These activities are accompanied by high emissions of greenhouse gases (GHG), and, in that respect, the International Maritime Organization (IMO) came up with a GHG strategy and reduction target in 2018. Almost 5 years later, the European Union (EU) introduced the FuelEU Maritime regulation to decrease the GHG intensity of fuels used by the EU maritime transport sector from 2% in 2025 to as much as 80% by 2050 as compared to the 2020 baseline (EU 2023). The effort to reduce GHG emissions is forcing the shipping industry to engage in technological innovation on a much larger scale than ever before, driving new advancements necessary to meet these stringent regulatory requirements. With the changing global shipping landscape, the need for technological innovation is high (Acciaro and Sys 2020). As a response, solutions such as zero-carbon fuels for shipping and engine improvements have been developed (Koukaki and Tei 2020; Bjerkan and Seter 2019).

Although plenty of such solutions have been developed, they are not adopted on a large scale. This is among others because they face several barriers related to high development costs, strict regulations, fierce global competition, and lack of financial incentives (Jia and Cui 2021; Wiegmans and Geerlings 2010). Innovation management scholars have suggested various factors for innovation adoption by drawing from various theories, including institutional theory (di Maggio and Powell 1983) and the Technology Acceptance Model (Davis 1989). However, they are primarily focused on business-to-consumer markets, and only a few attempts have been made to better understand factors for innovation adoption in a business-to-business market setting.

In this paper, we raise the question: what factors affect innovation adoption by ports? We answer that question by conducting a systematic literature review to develop a conceptual framework for innovation adoption by ports. More specifically, the Preferred Reporting Items for Systematic Reviews and Meta-Analyses (PRISMA) methodology was chosen (Page et al. 2021). Next, the principles of the thematic approach were used to cluster the identified factors into themes. This resulted in a comprehensive list of 17 factors clustered into 3 themes.

Through this effort, we contribute to the innovation management literature in several ways. It shows that factors for innovation adoption that have shown to be relevant in a business-to-consumer market are also applicable in a business-to-business market setting. Furthermore, this is the first time that factors for innovation adoption have been studied for the maritime sector. Our work has significant positive practical ramifications for the maritime sector as applying the factors will decrease uncertainty concerning innovation adoption for the stakeholders involved. We discuss how practitioners and public policymakers can apply the factors so that innovation adoption can be achieved by the sector.

## Literature background

Evolutionary economists stressed the cyclical nature of technological change and argued that technological discontinuities usher in periods of radical change up until the moment that a design becomes established as the dominant one (Anderson and Tushman 1990; Tushman and Murmann 1998; Tushman and Anderson 1986). That design impacts the strategies and market dynamics of various firms (Khazam and Mowery 1994). Scholars who focus on strategic management have come up with key factors that affect design dominance (Suarez 2004) such as choosing a proper point in time to enter the market and applying penetration pricing strategies, temporarily price below costs. Firms play a pivotal role as their strategic choices and investments influence which design becomes dominant. In the context of shipbuilding, a clear example of a dominant design is the steam engine-powered vessel in the nineteenth century, where sail-powered were replaced by steam-powered vessels. This shift resulted from a technological discontinuity (the steam engine) where steam-powered vessels rapidly became the dominant design in shipbuilding due to their superior speed, reliability, and range, regardless of wind conditions. Shipbuilding firms, recognizing these advantages, influenced and accelerated this shift by investing in steam technology, thus phasing out sailing ships. This example illustrates how a dominant design can emerge through a combination of technological innovation and strategic industry adaptation.

Innovation management scholars have, e.g., focused on how an individual or an organization adopts new technologies, ideas, or practices (Lai 2017). For example, according to Davis, users’ acceptance and adoption of new technologies are determined by the attitude of potential users toward technological innovation. He developed the Technology Acceptance Model (TAM) that distinguishes perceived usefulness and perceived ease of use of the technology as factors (Davis 1989). Perceived usefulness is defined as the potential user’s belief that using a particular system will improve their action, and perceived ease of use refers to the degree to which the likely user expects the target system to be free from effort (Davis 1989). In the last 3 decades, many authors have expanded the model to account for additional factors influencing technology adoption (Venkatesh and Davis 2000), (Venkatesh et al. 2003), (Venkatesh and Bala 2008). They have come up with factors such as subjective norms that refer to the belief that an important person or group of people will approve and support a particular behavior. They have also focused on the antecedents of perceived ease of use, incorporating anchoring and adjustment factors, as well as the moderating role of adopters (Venkatesh and Bala 2008).

Rogers (2003) distinguishes between five types of users that adopt technological innovation in different time periods. Innovators and early adopters will adopt an innovation first, followed by an early and a late majority. Finally, a group of laggards will adopt the innovation. He distinguishes five factors for innovation adoption: relative advantage, compatibility, complexity, trialability, and observability. Relative advantage is the degree to which an innovation is perceived as being better than the original or competing idea. The innovation must offer a significant incentive for the adopter to embrace the change, which can be expressed in terms of profit, time saved, social prestige, and usability. Second, compatibility refers to the extent to which the innovation is compatible with the firm regarding its infrastructure, cultural values, and social norms. Complexity is the degree to which an innovation is perceived as challenging to understand and use. Simple innovations are more likely to be adopted, whereas complex innovations might require extensive use of new tools, skills, and training. Fourth, trialability is the degree to which an innovation may be experimented with and plays a significant role in the early stages of innovation adoption. Finally, observability refers to the visibility of the innovation to the potential adopters.

In summary, evolutionary economists have described how dominant designs emerge in markets, while strategic management scholars study the factors that firms can apply to influence which design will reach dominance. Innovation management scholars zoom into the role that users of designs have and focus on how users accept and use technological innovation.

In addition, scholars that study maritime innovation have shown the existence of these innovations but also illustrate their non-adoption due to various constraints. For instance, it is known that digital tools and solutions can be used to increase operational efficiency of the maritime sector especially for global fleet monitoring, bunker fuel optimization or predictive vessel maintenance to name a few. However, it is often mentioned that financial constraints and/or a lack of technical expertise slowed the adoption of the innovations, particularly among smaller shipping companies that struggle with the high costs associated with implementing new technologies (Gavalas et al. 2022; Goerlandt and Pulsifer 2022).

The adoption of innovations in shipping also often relies on a network of interconnected actors, including but not limited to suppliers, business partners, and regulatory bodies. The adoption of one innovation is, thus, dependent on the issues of interoperability and stakeholder trust as it is the case for blockchain adoption in the maritime sector (Parola et al. 2020). Cultural resistance within shipping companies further impedes innovation adoption, as usual practices and a lack of training and skills development prevent new technologies from being fully utilized. Recognizing these factors are essential in helping the maritime industry especially the port sector to increase the adoption of technologies. A gap in the literature can be identified in that factors for innovation adoption have been haphazardly applied to the maritime sector, without coherence and structure, selecting a single or a small set at the authors’ discretion. By studying factors for innovation adoption mentioned in these literature streams and applying them to this specific sector, we study this gap in the literature.

## Methodology

To answer the research question of which factors affect innovation adoption by ports, we followed a two-stage approach: identification of factors and classification of factors. In the first stage, we applied the Preferred Reporting Items for Systematic Reviews and Meta-Analyses (PRISMA) methodology. The PRISMA methodology was chosen as it improves the transparency, adaptability, and replicability of the systematic review (Page et al. 2021). It consists of three steps for conducting a systematic review: (1) establishing a list of keywords to form a search query, (2) including or excluding articles based on predetermined criteria, and (3) examining the articles for relevance and identifying factors affecting innovation adoption by ports.

First, the search string was constructed. Four general keywords were identified by reviewing the research question: innovation, adoption, factors, and maritime. Subsequently, the paper’s authors organized a brainstorming session to investigate the synonyms of the keywords. Based upon that, an initial search string was constructed: ((Maritime OR Port OR ship* OR naval OR Marine) AND (Techno* OR Innovat*) AND (Adopt* OR Diffus* OR Acceptance OR Success) AND (Factor* OR Driver* OR Criteri* OR Determinant* OR Predictor* OR Attribute* OR Enabler* OR Cause* OR Antecedent*)). The choice for inclusion and exclusion criteria was discussed among all authors. The focus was on English studies that addressed the adoption of technological innovations by ports and not in other sectors. Both conceptual and empirical studies were selected, and literature reviews were excluded.

Second, the search string was applied to the Web of Science (WoS) database, and duplicates (11) were removed. The WoS offers reliable coverage of academic articles with complete citation linkages and metadata metrics. For this study, 1300 papers were identified from the WoS database.

Third, the first author read the titles and abstracts of papers, and relevant articles were selected for full reading. Papers whose abstracts mentioned that the paper studied innovation adoption by ports were fully read. One hundred eighty-six articles were found relevant, while one thousand one hundred twenty-one were excluded.

**Fig. 1:**
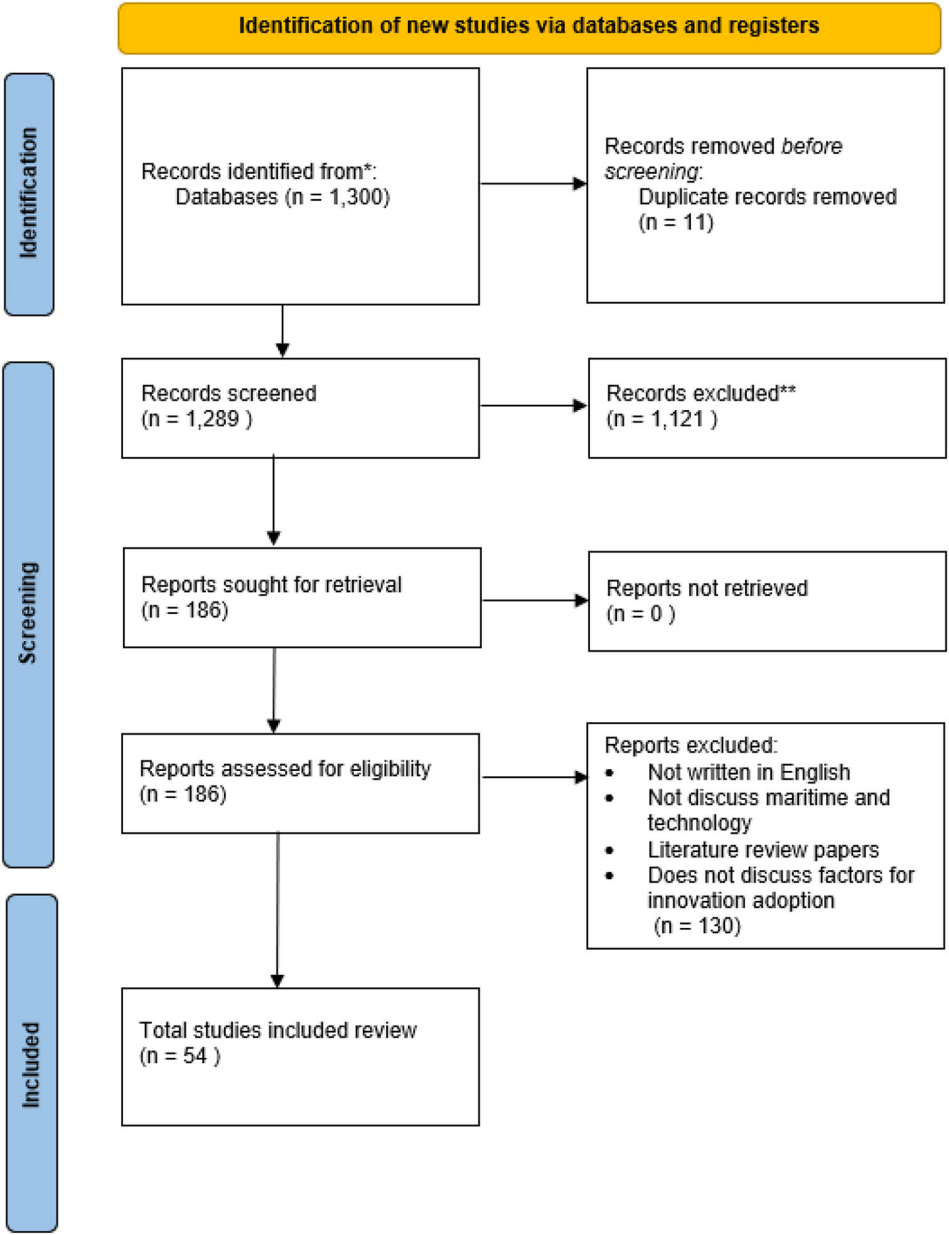
PRISMA flow diagram

We included a large number of articles at the beginning to make sure that we captured every study that might be relevant. Casting a broad net helped us avoid missing important research that could indirectly relate to innovation adoption in ports. This approach ensured that we took all literature into account, making sure no relevant factors were overlooked in the early stages of our review. For example, the inclusion of the term ‘port’ in the search query yielded a significant number of articles in the fields of electronic and computer science, as it is a widely used term in the information technology sector; these articles were excluded. In addition, papers that are not related to maritime technology, such as those on the marine ecosystem and biology, were excluded from the sample. Fourth, the 186 papers were fully read, and when they discussed factors for innovation adoption, they were taken into account. To evaluate the correctness of the exclusion criteria mentioned in Fig. 1, we have read several papers with potentially interesting titles and abstracts before exclusion from the analysis to assess the extent to which these papers were relevant. These papers were indeed correctly excluded from the analysis. This resulted in 54 articles. For every article found, the factors for innovation adoption were distilled. Factors were included in an Excel file when they were explicitly or implicitly mentioned. Figure 1 illustrates a schematic diagram of the strategy guiding the systematic literature review using PRISMA.

Subsequently, in the list of factors, duplicates were removed, and the remaining factors were clustered by the first and second authors, while the third author validated the results and categorization process. The factors were categorized using the principles of thematic analysis (Braun and Clarke 2006). The thematic analysis consists of (1) familiarization with the data (the list of factors retrieved from the literature review documents), (2) generation of initial codes, which we refer to as coded subgroups, (3) searching for themes among the coded subgroups, (4) reviewing the themes, (5) defining and naming themes, and (6) completing the report on the list of factors. The coded subgroups are the building blocks used to identify and categorize meaningful patterns within the data, while themes are the larger concepts that emerge from organizing and interpreting these codes.

Finally, synonyms were combined into unique factors where the starting point was a list of seven factors that have been discussed previously by innovation management scholars (see the literature background). These include relative advantage, complexity, compatibility, trialability, observability, perceived usefulness, and ease of use. Each new factor found in a paper was given a unique code if it did not match the first set of seven factors. Then the resulting coded factors were labeled and organized into three categories by the first and second authors. The third author reviewed the final list of themes for relevance.

## Results

Two hundred thirty-five potential factors were found. Using the method of thematic analysis, we have created 17 unique factors out of this list of potential factors. Subsequently, we analyzed these 17 factors for similarities and grouped them accordingly into 3 categories. The typology was developed using the thematic analysis and based on relevance to existing theories, frequency of mention in the literature, interrelationships among factors and feedback from one of the authors.

The factors are defined in Table 1. Each factor is explained in this table as well.

**Table 1:** Factors for a port’s innovation adoption

Category	Factor	Explanation
Adopting port’s characteristics	Know-how	The extent to which adopting ports have the expertise required to successfully implement the innovation. This relates to, e.g., the availability of technical skills (Doloreux 2006), the extent to which education and staff training (Zhou et al. 2020; Ren et al. 2018) is offered in the port, and prior experience (Acciaro et al. 2018; Vathanophas et al. 2008) with related innovations
	Organization support	The extent to which the firm’s management supports the prospective innovation (Zeng et al. 2021; Keceli et al. 2008; Tsai 2016; Yang and Lu 2012). When a port’s top management takes ownership and is committed to the innovation, the chances are higher that it will be adopted. For example, is there an innovation department responsible for evaluating and implementing innovations? Also, are the employees of these departments close to the port’s top management so that they can help increase organizational support
	Organizational structure	The capacity of the organization to coordinate the different steps needed to implement the innovation (Hermann and Wigger 2017; Arduino et al. 2013). When the port’s organizational structure is better, the chances are higher that the port will successfully adopt the innovation
	Financial capacity	The financial resources that are available for the port to adopt the innovation. This is related to the port’s size (Rey et al 2021; Keceli et al. 2008), its financial stability (Yuen et al. 2022), and availability of capital (Moldabekova et al. 2021; Zhou et al 2020)
	A port’s network embeddedness	The extent to which the port is embedded in a network of other stakeholders. The more the port collaborates with stakeholders (Doloreux 2006; Mosgaard & Kerndrup 2016), the higher the chances that the port will adopt the innovation because they can learn from each other. For example, when shippers participate in the network, the port authority can learn their preferred innovations and make choices accordingly
	Risk taking	The port’s stance toward taking risks. The willingness to take risks is dependent upon a port’s managerial and organizational culture. When it is more entrepreneurial, it will often take more risks (Moldabekova et al. 2021; Monteiro et al. 2013). The higher the willingness to take risks, the higher the chances that it will adopt the innovation
Characteristics of the innovation	Costs	The price of the innovation. The higher the price, the lower the chances that the port will adopt the innovation (Alahmadi et al., 2022; Yang & Lu 2012; Zeng et al. 2020; Gausdal et al. 2018)
	Relative advantage/perceived usefulness	The extent to which the innovation will bring advantage to the port when implemented. This advantage relates, e.g., to improvements related to environmental sustainability (Goerlandt & Pulsifer 2022; Bouman et al. 2017), profitability (Christodoulou and Cullinane 2019), efficiency (Gausdal et al 2018), competitive advantage (Doloreux 2006; Lee-Partridge et al. 2000), energy cost reductions (Christodoulou and Cullinane 2019), etc. The higher the relative advantage that the innovation brings, the higher the chances that it will be adopted
	Complexity/perceived ease of use	The extent to which it is easy to use the innovation. The more easy it is to use it, the higher the chances that the innovation will be adopted
	Compatibility	The extent to which the innovation can be integrated with other systems (Li and Yuen 2022; Yuen et al. 2022; Zeng et al. 2021, Bach et al 2020). The higher the compatibility, the higher the chances that the innovation will be adopted
	Trialability	The extent to which it is possible to try out an innovation (Mosgaard and Kerndrup 2016). When this is possible, the chances are higher that it will be adopted by the port
	Observability	The extent to which the firms are aware of the existence of a technological innovation. For example, is the firm aware of the benefits that the innovation will bring (Moldabekova et al. 2021)
Stakeholder pressures	Customer	Pressures from customers (such as ship owners) that act upon the port and make it more inclined to adopt the innovation (Lai et al. 2011). The higher these pressures, the higher the chances that the port will adopt the innovation
	Competitive port	Pressures from competing ports that act upon the port and make it more inclined to adopt the innovation (Zeng et al. 2021). A competing port that is more central to the focal port might choose to adopt an innovation and because of the fact that the port is its reputation in the international setting, other ports feel a pressure to adopt the same innovation. Therefore, the higher these pressure, the higher the chances that the port will adopt the innovation
	Regulatory body	Pressures from regulatory bodies (such as IMO) that act upon the port and make it more inclined to adopt the innovation (Fan et al 2022; Fonseca et al 2021; Henriquez et al. 2022; Raza 2020, Hermann and Wigger 2017, Vairetti et al. 2019; Wang et al. 2021; Wiśnicki et al., 2021). For example, when there is a strong interest from the state to adopt the innovation, ports will be more inclined to do so. Regulatory bodies may, e.g., make Subsidies and Tax Credits available. Furthermore, ports may have to comply with Industrial Norms (Lai et al. 2011), Standards (Arduino et al 2013), Harmonization of Indexes (Virto et al 2022). The higher these pressures, the higher the chances that the innovation will be adopted
	Societal	Pressures of society (such as NGOs) to adopt certain innovation (Martínez-Moya et al. 2019). The higher these pressures, the higher the chances that the innovation will be adopted

This section will present and elucidate the categories and factors. The first category is related to adopting port’s characteristics. This refers to all the characteristics of the adopting port that make it more inclined to adopt the innovation. This includes know-how, organizational support, organizational structure, financial capacity, a port’s network embeddedness, and the port’s stance toward risk-taking. Each presents a different classifiable element of the port, the combination of all these factors makes each port unique. Elements like organizational support and network embeddedness can even be time and subject dependent.

The second category is related to the characteristics of the innovation. It includes its costs, relative advantage and usefulness, complexity and perceived ease of use, compatibility, trialability, and observability. These factors are all linked to the current status of the innovation and can be both context and time dependent, e.g., costs can differ depending on time (development) and the situation in the port (existing infrastructure). In a similar way, perceived ease of use can change with time, but also experiences with other innovations or technologies can alter ones view.

The third category is stakeholder pressures, which include pressures coming from the customer, competitive ports, regulatory bodies, and societal pressures. There may be context-specific characteristics that can determine the strength and relevance of factors, but these are not included. For example, the specific country in which the firm is located may influence innovation adoption because of the existence of regional conditions, such as the existence of piracy (Perkovic et al., 2012) or rules related to when disasters happen, such as COVID-19 (Min 2022).

## Discussion and conclusion

This paper has studied factors for innovation adoption by ports. By conducting a thorough literature review, we come to a list of factors. The thematic analysis generated 3 themes from the extensive list of 203 unique factors. The themes consist of 17 factors. When we analyze these categories and factors, none of them relate to strategies that firms can apply to reach success of innovations they develop and promote. Instead, factors relate, e.g., to regulation. For example, stakeholders such as the regulatory bodies pressure the ports to adopt innovation. Therefore, we come to the conclusion that innovation in the maritime industry is not purely driven by competition but also by, e.g., regulation.

The topic of maritime innovation adoption is studied by multiple scholars with distinct scientific backgrounds. However, the journal Maritime Policy and Management has published the most contributions (4). That journal, together with the Journal of Cleaner Production, Sustainability, Energy Policy, International Journal of Shipping and Transport Logistics, Marine Pollution Bulletin, and Maritime Economics and Logistics, published 2 or more papers on the topic, while the remaining 36 papers were published in 36 separate publication outlets. Therefore, we conclude that apart from the journal Maritime Policy and Management, there are few single outlets that publish primarily on this topic. The 36 journals that publish only once on the topic are quite distinct. Therefore, there seems to be a diverse and interdisciplinary interest in innovation adoption by ports. Each journal addressed the subject from its unique disciplinary perspective. Although approaching this topic in a multidisciplinary manner can bring novel insights, there is also an inherent risk that if cross-fertilization does not take place and researchers from different disciplines do not work together, this can potentially result in duplication of work.

**Fig. 2:**
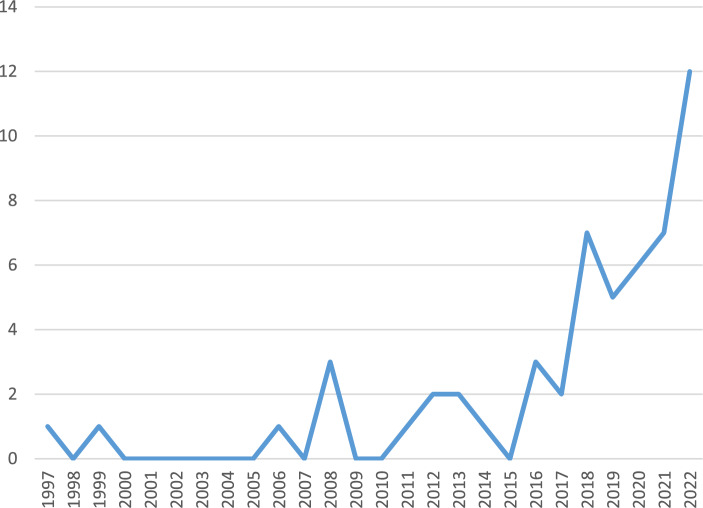
Articles per year

Figure 2 reports the number of articles published on innovation adoption per year. We can conclude that the interest in the topic of innovation adoption has risen over the years, especially since 2015. The trend demonstrates a growing research interest in technological innovation in the maritime sector compared to the previous decades. The topics of the studies after 2019 were primarily related to the acceptance of information systems (Li and Yuen 2022), port automation technology (Zeng et al. 2020), blockchain technology (Alahmadi et al. 2021; Zhou et al. 2020), and green ports and fuels (Chen et al. 2019). This surge in academic articles could be attributed to the pandemic effect, which has somehow augmented the digitalization and environmental trends in the maritime sector. The industry relies on technologies tested and adopted in other industries (Wiśnicki et al., 2021); a notable example is blockchain technology, which has already been extensively used in the banking sector (Alahmadi et al. 2021; Zhou et al. 2020).

### Contributions, limitations, and future research recommendations

This paper contributes to the literature in several ways. First, it contributes to evolutionary economics in general and the innovation management literature more specifically by showing that factors for innovation adoption can be distinguished and, therefore, the process of innovation adoption can be modeled. Second, it offers a list of factors that can be used by scholars to study innovation adoption by ports. The list of factors can be used by practitioners in the maritime setting to decrease uncertainty attached to the decision to adopt a technological innovation in that sector. Third, it studies factors for innovation adoption in a business-to-business context, whereas most papers that focus on innovation adoption focus on a business-to-consumer context. Finally, we provide a first indication that innovation adoption in the maritime setting is more driven by regulation than competition.

The research also has practical implications. For example, ports often face uncertainties regarding which innovations they should adopt. Our research is of benefit to them because it can be used to decrease the uncertainty attached to that decision for an innovation. Furthermore, the results can be used by public policymakers who might prefer a certain innovation to be adopted. They might utilize our research and influence innovation adoption factors so that their preferred innovation will succeed, thereby contributing to policy objectives.

This research is affected by some limitations. First, the research was limited to English-language articles. Research from other languages might have provided additional insights. Second, the focus lies on the WoS database, while other databases, such as Scopus and Google Scholar, were not included in this research. Future research could investigate non-English-language articles and articles in other databases to attempt to find additional factors for innovation adoption.

Future research could also investigate whether relations might exist between the factors identified in this paper. For example, it could be that the higher a port’s network embeddedness, the higher the know-how of that port because it can learn from the other ports with which it cooperates. Also, if the costs of the innovation are high, the port will be less inclined to take the risks attached to adopting the innovation.

Future research could also study whether weights for factors for innovation adoption can be established. In such a research setup, decision-makers at ports could be asked to compare the factors and assign preferences to them. Researchers who are interested in pursuing this can utilize multi-criteria decision-making methods such as the analytic hierarchy process (Saaty 1990) or the best–worst method (Rezaei 2015, 2016). Comparative studies could also be undertaken for different regions such as developed vs developing countries or global north vs global south. Finally, studies could explore whether innovation adoption by ports is affected more by regulation as opposed to competition.

## Data Availability

No datasets were generated or analyzed during the current study.
